# Neuropeptide Y Y_2_ receptors modulate trace fear conditioning and spatial memory in the dorsal hippocampus

**DOI:** 10.1186/1471-2210-11-S2-A2

**Published:** 2011-09-05

**Authors:** Ramon O Tasan, Dilip Verma, Mario Mietzsch, Stefan Weger, Regine Heilbronn, Herbert Herzog, Günther Sperk

**Affiliations:** 1 Institute of Pharmacology, Medical University Innsbruck, 6020 Innsbruck, Austria; 2 Institute of Virology, Charité, Campus Benjamin Franklin, Free University of Berlin, 12203 Berlin, Germany; 3 Neuroscience Research Program, Garvan Institute of Medical Research, Darlinghurst NSW 2010, Australia

## Background

Neuropeptide Y (NPY), a highly conserved 36 amino acid peptide is widely distributed in the central nervous system. Besides its functions in various metabolic processes NPY has attracted considerable attention in modulating emotional-affective behavior. NPY exerts a pronounced anxiolytic effect most likely mediated by Y_1_ receptors, whereas stimulation of predominantly pre-synaptic Y_2_ receptors results in increased anxiety. The role of NPY Y_2_ receptors in the processing of emotional learning, however, remains still elusive.

## Methods

The current study aims to investigate the role of NPY Y_2_ receptors in Pavlovian fear conditioning, a simple form of associative learning and in a spatial memory task, the Barnes maze. Y_2_-KO mice were subjected to delay (amygdala-dependent) and trace (hippocampus-dependent) fear conditioning paradigms.

## Results

While in delay fear conditioning Y_2_-KO mice performed similar to wild-type controls, recall of a trace fear memory was significantly increased in Y_2_-KO mice. Furthermore, Y_2_-KO mice exhibited an improved long-term memory in the Barnes maze test, a paradigm investigating spatial learning. Trace fear conditioning and spatial memory are predominantly mediated by the dorsal hippocampus. For investigating the specific contribution of Y_2_ receptors in the adult dorsal hippocampus in trace fear conditioning and spatial memory formation we locally deleted hippocampal Y_2_ receptors in conditional Y_2_-KO mice by injection of a rAAV-CreGFP vector. Moreover we over-expressed NPY_3–36_, an Y_2_ receptor preferring agonist, at the same brain sites.

## Conclusions

Our data indicate that while Y_2_ receptors are not involved in amygdala-dependent delay fear conditioning, they seem to play an inhibitory role on the acquisition of trace fear memories. Moreover, Y_2_ receptors in the dorsal hippocampus are crucial for spatial memory formation. These actions are probably mediated by inhibition of glutamate release in dorsal hippocampal circuitries.

